# Early versus later response to treatment in patients with community-acquired pneumonia: analysis of the REACH study

**DOI:** 10.1186/1465-9921-15-6

**Published:** 2014-01-22

**Authors:** Francesco Blasi, Helmut Ostermann, Jill Racketa, Jesús Medina, Kyle McBride, Javier Garau

**Affiliations:** 1 Department of Pathophysiology and Transplantation, University of Milan, IRCCS Fondazione Ospedale Maggiore, Policlinico Cà Granda Milano, Italy; 2 Department of Internal Medicine III, Haematology and Oncology, University Hospital Munich, Munich, Germany; 3 Health Economics and Outcomes Research, AstraZeneca, 1800 Concord Pike, 19850 Wilmington, DE, USA; 4 Medical Evidence Centre (Global Medical Affairs), AstraZeneca, Parque Norte, Edificio Roble, Serrano Galvache 56, 28033 Madrid, Spain; 5 Instat Services, Inc., 1 Wilson Street, Chatham, NJ 07928 USA; 6 Department of Medicine, Hospital Universitari Mutua de Terrassa, Plaza Doctor Robert 5, 08221 Terrassa, Barcelona, Spain

**Keywords:** Clinical stability, Community-acquired pneumonia, Early response, Resource use

## Abstract

**Background:**

Key goals in the treatment of CAP include early response to treatment and achievement of clinical stability. The US FDA recommends early response endpoints (72 hours after initiation of treatment) in clinical trials for the treatment of community-acquired bacterial pneumonia. REACH (REtrospective Study to Assess the Clinical Management of Patients With Moderate-to-Severe Complicated Skin and Soft Tissue Infections [cSSTI] or CAP in the Hospital Setting) was a retrospective observational study, providing current data on the clinical management and resource burden of CAP in real-life settings in European hospitals. This analysis reviews the characteristics and outcomes of patients showing early positive response to treatment (time to clinical stability [TCS] ≤4 days, as assessed by Halm’s criteria) compared with patients with later positive response (TCS >4 days).

**Methods:**

Patients were adults, hospitalized with CAP (2010–2011) and requiring in-hospital treatment with intravenous antibiotics.

**Results:**

Of the 2039 patients included in REACH, 585 (28.7%) had TCS assessed by Halm’s criteria: 332 (56.8%) showed early response (median 3.0 days), and 253 (43.2%) showed later response to treatment (median 7.0 days). Use of Halm’s criteria varied across participating countries, ranging from 0% (Belgium) to 49.1% (UK). Patient characteristics and relevant medical history were similar between the two groups. There were no notable differences in initial antibiotic therapy between groups, except that more early responders had been treated with amoxicillin–clavulanate and amoxicillin monotherapy (22.6%; 7.5%, respectively) than later responders (5.9%; 1.2%, respectively). Initial treatment modification and re-infection or recurrences were less frequent in early responders compared with later responders (14.2% and 3.3% vs. 34.8% and 5.9%, respectively). Early responders had a shorter duration of hospitalization (mean 9.4 ± SD 7.0; median 8.0 days vs. mean 15.6 ± SD 10.5; median 12.0 days, respectively), lower rate of ICU admission (3.3% vs. 21.3%) and shorter duration of ICU stay (mean 6.2 ± SD 5.7; median 4.0 days vs. mean 10.4 ± SD 10.1; median 8.0 days, respectively) compared with later responders. Mortality was low in both groups.

**Conclusions:**

Achieving early clinical stabilization in CAP (≤4 days) is associated with improved outcomes, lower requirement for initial treatment modification or readmission and lower resource use, compared with a later response.

**Trial registration:**

NCT01293435

## Introduction

In Europe, the clinical and economic burden of community-acquired pneumonia (CAP) remains high [1], and hospitalization for CAP is responsible for up to 80% of the total costs of this disease [2]. REACH (REtrospective Study to Assess the Clinical Management of Patients With Moderate-to-Severe Complicated Skin and Soft Tissue Infections [cSSTI] or CAP in the Hospital Setting) was a retrospective, observational study undertaken in 128 centres in 10 European countries, funded by AstraZeneca, and designed to provide current data on the clinical management and resource burden of CAP in real-life settings in hospitals [3]. The data obtained from this study reveal a large heterogeneity in clinical management patterns. Initial antibiotic treatment modification, for any reason, occurred in around one-third of affected patients and was more common in patients with comorbidities than in those without. Rates of initial antibiotic treatment modification and associated outcomes, such as overall mortality, were increased in patients with more complicated or more severe illness and were associated with considerable additional resource use, particularly length of hospital stay (Ostermann *et al*., REACH CAP health economics publication). A number of other factors influence patient outcomes in CAP, such as the decision whether to hospitalize [4,5], as patients with low-risk disease fare better with outpatient treatment [5] and the greatest proportion of pneumonia-related mortality and healthcare expenditure occurs among those persons who are hospitalized [5-7]. This decision is often based on severity assessment and the presence of risk factors associated with severe CAP, including age (≥ 65 years), nursing home residence and comorbidities such as congestive heart failure, chronic renal failure and diabetes mellitus [1,8-10], and recurrent infections may affect outcomes. There is often difficulty in obtaining a microbiological diagnosis and the pathogen is not identified in a high proportion (35–67%) of cases, resulting in empiric rather than targeted antibacterial treatment [11,12]. Early failure of treatment (within 48–72 hours) is associated with high morbidity and mortality rates and most cases occur because of inadequate response [13]. The monitoring of response to treatment, as recommended by The European Respiratory Society (ERS), in collaboration with The European Society for Clinical Microbiology and Infectious Diseases (ESCMID) by simple clinical criteria, including body temperature, respiratory and haemodynamic parameters, is therefore essential [4].

In clinical practice, clinical stability is acknowledged as a guide to when antibiotic therapy may be switched from intravenous (IV) to oral. Thus, the recognition of clinical stability is important as an outcome for treatment of CAP. The guidelines on the management of CAP published by the American Thoracic Society (ATS) and the Infectious Diseases Society of America (IDSA) [5] give a definition of clinical stability that is based on objective parameters and developed from those described by Halm *et al*. [14]. These criteria were developed as a specific and objective measure for clinical stability in CAP and are consistent with, and similar to, recent US Food and Drug Administration (FDA) recommendations for early response endpoints (72 hours after initiation of treatment) in non-inferiority clinical trials for the treatment of community-acquired bacterial pneumonia [15]. This recommendation resulted from a consultation of the Anti-Infective Drugs Advisory Committee and was largely based on historical data [15]. This endpoint may be more clinically relevant, as an early indication of treatment failure can allow the selection of an alternative antimicrobial therapy and avoid prolonged use of inappropriate treatment, which may impact clinical outcomes and lower healthcare resource utilization.

The purpose of this subanalysis of the REACH study was to analyse the characteristics of patients showing an early response to treatment (time to clinical stability [TCS] ≤ 4 days) compared with patients with later response (TCS > 4 days) and to identify any impact on associated outcomes.

## Methods

REACH was a multinational, multicentre, observational, retrospective cohort study of patients hospitalized with CAP (NCT01293435). A total of 2,039 patients, aged 18 years or older, hospitalized and treated with IV antibiotics were enrolled in 128 sites in 10 participating countries (Belgium, France, Germany, Greece, Italy, the Netherlands, Portugal, Spain, Turkey and the UK). The study was performed according to Good Clinical Practice and the Declaration of Helsinki. All local ethics committees approved the study protocol. Local legislation relating to written informed consent for non-interventional studies was followed in each country; in Germany and Portugal, where this information is mandatory, written informed consent was collected. The list of ethics committees and health authorities that approved the study is available to the public at: http://www.astrazenecaclinicaltrials.com. All patients included in the analysis were hospitalized between March 2010 and February 2011. Study design and patient inclusion and exclusion criteria are summarized in the REACH primary publication [3]. In brief, the study collected data regarding patient demographics, disease characteristics and diagnosis, management, clinical outcomes (including modification of initial antibiotic treatment for any reason and TCS) and healthcare resource use, via an electronic Case Report Form (eCRF) completed by the investigator at each site.

‘Initial antibiotic treatment modification’ was defined as a change in initial antibiotic treatment due to insufficient response, adverse reaction, interaction with other drugs, non-suitability of the initial antibiotic based on the results of microbiological tests, or changes to or additions of new agents in a subsequent line (alone or in combination). The eCRF also allowed a response of ‘other’ or ‘unknown’. Several antibiotic treatment modifications in the same patient were counted as a single case. Changes in dose or frequency of an existing antibiotic (considered as dose escalation or adaptation), removal of an antibiotic from a combination and adaptation of the dose or frequency of the remaining antibiotic, were not considered as treatment modification. TCS was recorded on the eCRF in response to the question ‘What was the time to clinical stability?’ Available options were ‘based on the Halm criteria for clinical stability’ (specifically, the first day that the following clinical variables are stable: systolic blood pressure ≥ 90 mm Hg; heart rate ≤ 100 beats/min; respiratory rate ≤ 24; temperature ≤ 38.3°C; oxygen saturation ≥ 90%; ability to eat; normal mental function) [14]. Alternative options were ‘based on the change from IV to oral therapy’, ‘based on other criteria’ and ‘other’.

This descriptive analysis focuses on the characteristics, antibiotic treatments, clinical outcomes and use of healthcare resources of those patients from the REACH study whose achievement of clinical stability was assessed according to Halm’s criteria [14]. Characteristics of patients showing an early response to treatment (defined as a TCS ≤ 4 days) were compared with those of patients with a later response (TCS > 4 days), and corresponding outcomes and resource use were measured. A further analysis was conducted of patients who died in both the early and late responders groups.

## Results

### Use of Halm’s criteria for the assessment of clinical stability in the participating countries

Of the 2039 patients included in the REACH study, 585 (28.7%) patients had TCS assessed by Halm’s criteria [14], as noted on the eCRF. The use of Halm’s criteria was variable across the different participating countries; Belgium (n = 0/191; 0%); France (n = 122/366; 33.3%); Germany (n = 7/50; 14%); Greece (n = 71/215; 33.0%); Italy (n = 91/300; 30.3%); the Netherlands (n = 39/203; 19.2%); Portugal (n = 13/121; 10.7%); Spain (n = 131/279; 47.0%); Turkey (n = 55/200; 27.5%) and UK (n = 56/114; 49.1%).

### Patient population

Of the 585 patients who had TCS assessed by Halm’s criteria [14], 332 (56.8%) showed early response (≤ 4 days; median 3.0 days) and 253 (43.2%) showed later response to treatment (> 4 days; median 7.0 days). Patient demographics were similar between the two groups, although some numerical differences were observed (Table 1). Disease characteristics and triggering symptoms are shown in Table 2. Patients without comorbidities tended to be older in the later responders group (mean age 55.5 years vs. 51.9 years in the early responders group). Fewer of the early responders had comorbidities compared with the later responders (respiratory disease 32.5% vs. 35.6%; diabetes 16.3% vs. 20.6%; congestive heart disease 14.5% vs. 20.2%, respectively) (Table 2). Disease severity, determined by PORT/PSI (Pneumonia Outcomes Research Team/Pneumonia Severity Index) [4,16] or CURB-65 (Confusion, Urea nitrogen, Respiratory rate, Blood pressure, ≥ 65 years of age) [4] scores, indicates that later responders tended to have more severe disease (PORT/PSI class V) compared with early responders (18.2% vs. 7.7% of patients with a PORT/PSI score, respectively). Interestingly, numerically more later responders had a PORT/PSI or CURB-65 score recorded than patients with an early response (30.4% vs. 19.6%; 46.6% vs. 38.9%, respectively) (Table 3).

**Table 1:** Patient demographics

**Characteristic, n (%)**	**Early responders n = 332**	**Later responders n = 253**	**Patients without Halm-based assessment of TCS n = 1454**
Age, years, mean (SD) [median]	63.8 (19.0) [68]	64.5 (18.2) [69]	64.7 (18.5) [68]
≥ 65 years	187 (55.8)	137 (54.2)	827 (56.9)
Sex, male	204 (61.4)	144 (56.9)	848 (58.3)
Ethnic origin			
White	225 (67.8)	217 (85.8)	1131 (77.8)
Non-white	8 (2.4)	3 (1.2)	40 (2.8)
Unknown/missing	9 (2.7)	3 (1.2)	39 (2.7)
Not applicable*	90 (27.1)	30 (11.9)	244 (16.8)
Previous admission to hospital with CAP (last 3 months)	8 (2.4)	9 (3.6)	82 (5.6)
Haemodialysis	0	1 (0.4)	5 (0.3)
Chemotherapy for active cancer	1 (0.3)	1 (0.4)	28 (1.9)
Smoking status^†^			
Non-smoker	129 (38.5)	108 (42.7)	467 (32.1)
Ex-smoker	94 (28.1)	79 (31.2)	380 (26.1)
Occasional smoker	2 (0.6)	2 (0.8)	38 (2.6)
Habitual smoker	89 (26.8)	46 (18.2)	328 (22.6)
Unknown	18 (5.4)	18 (7.1)	241 (16.6)

*All patients in this category were from France, where this question is not permitted in clinical studies.

^†^Definitions: Never smoked: patients who have never smoked > 20 g of tobacco in their lifetime; Ex-smoker: patients who stopped smoking ≥ 365 days ago: Occasional smoker: patients who smoke < 1 tobacco product per day; Habitual smoker: patients who smoke ≥ 1 tobacco products/day.

SD = standard deviation; TCS = time to clinical stability.

**Table 2:** Medical history and disease characteristics

**Characteristic**	**Early responders n = 332**	**Later responders n = 253**	**Patients without Halm-based assessment of TCS n = 1454**
Relevant medical conditions at hospitalization (initial visit*) (≥ 5% of analysis population), n (%)			
Any relevant condition	255 (76.8)	210 (83.0)	1133 (77.9)
Respiratory disease	108 (32.5)	90 (35.6)	491 (33.8)
Diabetes	54 (16.3)	52 (20.6)	263 (18.1)
Congestive heart disease	48 (14.5)	51 (20.2)	237 (16.3)
Cancer/malignancy	30 (9.0)	27 (10.7)	180 (12.4)
Peripheral vascular disease	34 (10.2)	23 (9.1)	126 (8.7)
Renal disease	17 (5.1)	20 (7.9)	110 (7.6)
Other relevant conditions^†^	118 (35.5)	102 (40.3)	464 (31.9)
Age of patients with comorbidities, years, mean (SD) [median]	67.5 (17.2) [71]	66.3 (17.92) [72]	68.2 (16.4) [71]
Age of patients without comorbidities, years, mean (SD) [median]	51.9 (19.7) [49]	55.5 (17.0) [54]	52.4 (20.1) [50]
Medication history in the 3 months prior to hospitalization, n (%)			
Any prior medication	159 (47.5)	148 (58.5)	836 (57.5)
Antibiotics/antivirals	51 (15.4)	52 (20.6)	292 (20.1)
Anticoagulants	56 (16.9)	39 (15.4)	206 (14.2)
Immunosuppressants/immunomodulators	19 (5.7)	19 (7.5)	113 (7.8)
Non-steroidal anti-inflammatory drugs	23 (6.9)	16 (6.3)	98 (6.7)
Any other relevant therapies*	58 (17.5)	47 (18.6)	274 (18.8)
Unknown	8 (2.4)	18 (7.1)	120 (8.3)

*Visit to hospital for current infection or date of diagnosis of infection for patients already hospitalized.

^†^As defined by the investigator.

SD = standard deviation; TCS = time to clinical stability.

**Table 3:** Characteristics of index community-acquired pneumonia infection

**Characteristic, n (%)**	**Early responders n = 332**	**Later responders n = 253**	**Patients without Halm-based assessment of TCS n = 1454**
Type of CAP			
CAP*	279 (84.0)	210 (83.0)	1118 (76.9)
HCAP^†^	24 (7.2)	20 (7.9)	201 (13.8)
Immunocompromised/immunosuppressed	8 (2.4)	11 (4.3)	53 (3.6)
Other	0	2 (0.6)	37 (2.5)
Unknown	21 (6.3)	10 (4.0)	54 (3.7)
Radiographic findings suggestive of bacterial pneumonia			
Infiltrate	175 (52.7)	113 (44.7)	880 (60.5)
Consolidation	183 (55.1)	167 (66.0)	597 (41.1)
Pleural effusion	40 (12.0)	44 (17.4)	235 (16.2)
Other	19 (5.7)	14 (5.5)	67 (4.6)
Unknown	0	1 (0.4)	15 (1.0)
Signs of acute illness at diagnosis			
New or increased cough	258 (77.7)	202 (79.8)	1115 (76.7)
Purulent sputum or change in sputum character	182 (54.8)	127 (50.2)	744 (51.2)
Auscultatory findings consistent with pneumonia	253 (76.2)	184 (72.7)	1055 (72.6)
Dyspnoea, tachypnoea or hypoxaemia	256 (77.1)	210 (83.0)	1025 (70.5)
Fever or hypothermia	213 (64.2)	153 (60.5)	951 (65.4)
White blood cell count > 10000 cells/mm^3^ or < 4500 cells/mm^3^	210 (63.3)	179 (70.8)	963 (66.2)
Prognosis based on severity indices			
PORT/PSI	65 (19.6)	77 (30.4)	212 (14.6)
I/II^‡^	17 (26.2)	13 (16.9)	41 (19.3)
III/IV	43 (66.2)	50 (64.9)	138 (65.1)
V	5 (7.7)	14 (18.2)	33 (15.6)
CURB-65	129 (38.9)	118 (46.6)	280 (19.3)
0-1^‡^	49 (38.0)	23 (19.5)	82 (29.3)
2	38 (29.5)	42 (35.6)	105 (37.5)
3	16 (12.4)	23 (19.5)	61 (21.8)
4	21 (16.3)	26 (22.0)	21 (7.5)
5	2 (1.6)	3 (2.5)	3 (1.1)
Unknown	3 (2.3)	1 (0.8)	8 (2.9)

*Residence in private house or apartment only.

^†^Responses considered HCAP were all other residential statuses, with the exception of immunocompromised/immunosuppressed.

^‡^The proportions are calculated with respect to the total number of patients in which this score was reported to have been used to assess clinical stability.

CAP = community-acquired pneumonia; CURB-65 = Confusion, Urea nitrogen, Respiratory rate, Blood pressure, ≥ 65 years of age; HCAP = healthcare-associated pneumonia; PORT/PSI = Pneumonia Outcomes Research Team/Pneumonia Severity Index; SD = standard deviation; TCS = time to clinical stability.

### Diagnostic information

All patients underwent a microbiological test, the majority having sputum examination (39.5% early responders and 47.0% later responders) and/or blood culture (49.4% early responders and 60.1% later responders). Negative cultures were more frequent in early (74.7%) than in later responders (66.0%) (Table 4). In the patients with a microbiologically confirmed infection (n = 87 in both groups), early responders were less frequently infected with ‘difficult-to-treat’ microorganisms, such as *Pseudomonas aeruginosa* (4.6%), or had aspiration pneumonia (1.1%), compared with later responders (9.2% and 4.6%, respectively). There was a single case of methicillin-resistant *Staphylococcus aureus* detected in a later-responder. Other microorganisms were found in similar proportions in both subpopulations.

**Table 4:** Microbiological diagnosis

**Microbiological diagnosis, n [patients with a positive microbiological diagnosis] (%)**	**Early responders n = 332**	**Later responders n = 253**	**Patients without Halm-based assessment of TCS n = 1454**
Number of patients with a microbiological diagnosis	87 (26.2)	87 (34.4)	408 (28.1)
Gram-positive cocci*	40 (46.0)	38 (43.7)	208 (51.0)
*Streptococcus pneumoniae*	32 (36.8)	28 (32.2)	168 (41.2)
Penicillin-resistant *Streptococcus pneumoniae*	0	1 (1.1)	1 (0.2)
Methicillin-resistant *Staphylococcus aureus*	0	1 (1.1)	11 (2.7)
Methicillin-susceptible *Staphylococcus aureus*	9 (10.3)	9 (10.3)	33 (8.1)
*Legionella* spp.	4 (4.6)	3 (3.4)	13 (3.2)
*Mycoplasma pneumoniae*	4 (4.6)	1 (1.1)	13 (3.2)
*Chlamydophila pneumoniae*	0	2 (2.3)	11 (2.7)
Gram-negative bacilli^†^	15 (17.2)	11 (12.6)	81 (19.8)
*Haemophilus influenzae*	7 (8.0)	3 (3.4)	23 (5.6)
*Haemophilus parainfluenzae*	3 (3.4)	1 (1.1)	2 (0.5)
*Moraxella catarrhalis*	2 (2.3)	0	7 (1.7)
*Pseudomonas aeruginosa*	4 (4.6)	8 (9.2)	29 (7.1)
*Escherichia coli*	4 (4.6)	3 (1.4)	29 (7.1)
*Klebsiella* spp.	2 (2.3)	0	17 (4.2)
Aspiration pneumonia^§^	1 (1.1)	4 (4.6)	12 (2.9)
Other microorganisms	19 (21.8)	32 (36.8)	82 (20.1)
Unknown (not identified)	248 (74.7)	167 (66.0)	1060 (72.9)

*Includes other *Streptococcus* spp. and *Staphylococcus* spp.

^†^Includes *Morganella morganii*, *Pasteurella multocida, Proteus mirabilis *and *Serratia marcescens, Acinetobacter* spp. and *Hafnia alvei*.

^§^Commonly applied to situations when a patient with risk factors for aspiration presents with pneumonia.

### Outcomes and resource use

No notable differences in first-line antibiotic therapy were seen between groups, except that amoxicillin–clavulanate or amoxicillin monotherapy as initial therapy was more commonly used in early responders (22.6% and 7.5%, respectively) than later responders (5.9% and 1.2%, respectively) (Table 5).

**Table 5:** Antibiotic therapies

**Antibiotic, n (%)**	**Early responders n = 332**	**Later responders n = 253**
Combinations		
Penicillin or penicillin + β-lactamase inhibitor + macrolide	50 (15.1)	22 (8.7)
Cephalosporin (except cefuroxime) + macrolide	33 (9.9)	48 (19.0)
Cephalosporin (except cefuroxime) + fluoroquinolone	25 (7.5)	34 (13.4)
Penicillin or penicillin–β-lactamase + fluoroquinolone	16 (4.8)	17 (6.7)
Monotherapies		
Amoxicillin–clavulanate	75 (22.6)	15 (5.9)
Amoxicillin	25 (7.5)	3 (1.2)
Ceftriaxone	25 (7.5)	16 (6.3)
Levofloxacin	22 (6.6)	16 (6.3)
Moxifloxacin	15 (4.5)	10 (4.0)
Ampicillin–sulbactam	5 (1.5)	3 (1.2)
Piperacillin–tazobactam	4 (1.2)	7 (2.8)
Azithromycin	4 (1.2)	3 (1.2)
Clarithromycin	3 (0.9)	3 (1.2)
Cefuroxime	3 (0.9)	2 (0.8)
Meropenem	1 (0.3)	2 (0.8)
Ciprofloxacin	0	2 (0.8)

The most notable difference in outcomes was that early responders had a lower requirement for initial antibiotic treatment modification (14.2%) than patients with a later response (34.8%). Readmission to hospital after discharge was also less frequently observed in early responders than in later responders (Table 6).

**Table 6:** Clinical outcomes and hospital resource use

**Outcome, n (%)**	**Early responders n = 332**	**Later responders n = 253**	**Patients without Halm-based assessment of TCS n = 1454**
Antibiotic treatment modification while on initial therapy (excluding streamlining*)	47 (14.2)	88 (34.8)	454 (31.2)
Time to clinical stability, days, mean (SD) [median]	2.6 (0.95) [3.0]	9.0 (6.53) [7.0]	5.7 (5.0) [5.0]
Length of stay^†^, days, mean (SD) [median]	9.4 (7.0) [8.0] (n = 309)	15.6 (10.5) [12.0] (n = 246)	12.6 (10.7) [10.0] (n = 1423)
Length of stay after achievement of clinical stability^‡^, days, mean (SD) [median]	5.9 (7.1) [4.0] (n = 305)	5.9 (7.0) [3.0] (n = 231)	N/A
Patients admitted to ICU	11 (3.3)	54 (21.3)	213 (14.6)
Time in ICU^‡^, days, mean (SD) [median] (n)	6.2 (5.7) [4.0] (n = 11)	10.4 (10.1) [8.0] (n = 53)	9.5 (12.4) [5.0]
Patients admitted to ICU after achievement of clinical stability	0	1 (0.4)	N/A
Reinfection/recurrence^§^	11 (3.3)	15 (5.9)	68 (4.7)
Home-based care after discharge	6 (1.8)	11 (4.3)	56 (3.9)
Blood pressure support during hospitalization			
Fluid resuscitation	24 (7.2)	37 (14.6)	190 (13.1)
Vasopressors	2 (0.6)	17 (6.7)	82 (5.6)
Invasive procedures	1 (0.3)	3 (1.2)	26 (1.8)
Isolation required	28 (8.4)	24 (9.5)	102 (7.0)
Mechanical ventilation required during hospitalization	24 (7.2)	48 (19.0)	208 (14.3)
Invasive	12 (3.6)	28 (11.1)	99 (6.8)
Non-invasive	12 (3.6)	25 (9.9)	129 (8.9)
Parenteral nutrition	5 (1.5)	19 (7.5)	70 (4.8)
Duration of parenteral nutrition, days, mean (SD) [median]	6.0 (3.5) [5.0] (n = 5)	8.2 (5.1) [6.5] (n = 18)	9.5 (12.0) [5.0] (n = 65)
Acute renal failure necessitating renal replacement therapy	1 (0.3)	6 (2.4)	39 (2.7)
Duration of renal failure, days, mean (SD) [median]	2.0 (0) [2.0] (n = 1)	14.3 (16.0) [9.5] (n = 4)	5.7 (7.2) [3.0] (n = 32)
Septic shock during hospitalization	1 (0.3)	20 (7.9)	63 (4.3)
Mortality	2 (0.6)	4 (1.6)	141 (9.7)
Post-discharge mortality	0	0	0

*De-escalation of treatment to narrower-spectrum antibiotics upon patient improvement or confirmed microbiological diagnosis.

^†^Includes duration of all hospitalizations for patients with recurrences.

^‡^Does not include duration of all hospitalizations for patients with recurrences.

^§^Refers to patients hospitalized again (due to CAP) after initial discharge.

ICU = intensive care unit; N/A = not applicable; SD = standard deviation.

An association between early response and shorter duration of hospitalization (mean 9.4; standard deviation [SD]: 7.0; median 8.0 days), lower rate of admission to the intensive care unit (ICU; 3.3%) and shorter duration of ICU stay (mean 6.2; SD: 5.7; median 4.0 days) was observed compared with later responders (mean 15.6; SD: 10.5; median 12.0 days; 21.3%; mean 10.4; SD: 10.1; median 8.0 days, respectively).

Hospital resource use, such as blood pressure support, mechanical ventilation and parenteral nutrition, was higher in patients who had a later response, and there were more cases of septic shock compared with patients with an early response (7.9% vs. 0.3%) (Table 6). There were few deaths reported (n = 6). Reasons for death in early responders (n = 2) were CAP-related in one case, and non-CAP-related in the other, based on investigator assessment. Death in later responders (n = 4) was related to CAP in two cases, unrelated in one and unknown in the fourth. “Post-clinical stability”, differences between early and later responders in terms of length of stay and ICU admissions were minimal (mean length of hospital stay 5.9 days, both early and later responders (Table 6).

A comparison of patient characteristics for those patients who did not have TCS assessed by Halm’s criteria revealed no relevant differences in the baseline data, apart from a small difference in the proportion of patients with healthcare-associated pneumonia (HCAP) (13.8% vs. 7.2% of early responders and 7.9% of later responders). In addition, a smaller proportion of patients had PORT/PSI or CURB-65 assessment of disease severity (Table 3). Clinical outcomes and resource use were also similar, except for a higher mortality rate observed in patients not assessed by Halm’s criteria (9.7% vs. 0.6% for early responders and 1.6% for later responders) (Table 6).

An analysis of those patients who had received antibiotic pre-treatment, compared with those who had not, showed that pre-treatment with antibiotics was associated with a shorter hospital length of stay for both early and later responders (mean 8.6 vs. 10.1 days for early responders and 15.3 vs. 17.0 days for later responders) (Table 7). Fewer later responders with antibiotic pre-treatment were admitted to ICU, and those that were, remained there for a shorter duration than patients without antibiotic pre-treatment. A higher mortality rate was observed in late responders with antibiotic pre-treatment compared with those without pre-treatment (5.2% vs. 0.5%) (Table 7).

**Table 7:** Clinical outcomes and hospital resource use for patients with and without antibiotic pre-treatment

**Outcome, n (%)**	**Early responders n = 332**		**Later responders n = 253**	
	**Without antibiotic pre-treatment n = 266**	**With antibiotic pre-treatment n = 61**	**Without antibiotic pre-treatment n = 184**	**With antibiotic pre-treatment n = 58**
Antibiotic treatment modification while on initial therapy (excluding streamlining*)	38 (14.3)	7 (11.5)	66 (35.9)	20 (34.5)
Time to clinical stability, days, mean (SD) [median] based on Halm's criteria	2.6 (1.0) [2.0]	2.8 (0.9) [3.0]	9.2 (7.0) [7.0]	8.5 (5.7) [7.0]
Length of stay^†^, days, mean (SD) [median]	10.1 (7.7) [8.0]	8.6 (4.5) [7.0]	17.0 (12.6) [13.0]	15.3 (9.9) [12.0]
Patients admitted to ICU	9 (3.4)	2 (3.3)	44 (23.9)	9 (15.5)
Time in ICU^‡^, days mean (SD) [median] (n)	6.3 (6.3) [4.0] (n = 9)	5.5 (2.1) [5.5] (n = 2)	10.8 (10.9) [8.0] (n = 44)	8.9 (4.3) [9.0] (n = 8)
Reinfection/recurrence^§^	9 (3.4)	2 (3.3)	10 (5.4)	5 (8.6)
Home-based care after discharge	6 (2.3)	0	9 (4.9)	2 (3.4)
Blood pressure support during hospitalization				
Fluid resuscitation	19 (7.1)	4 (6.6)	26 (14.1)	9 (15.5)
Vasopressors	2 (0.8)	0	12 (6.5)	5 (8.6)
Invasive procedures	0	1 (1.6)	3 (1.6)	0
Isolation required	24 (9.0)	3 (4.9)	17 (9.2)	7 (12.1)
Mechanical ventilation required during hospitalization	23 (8.6)	1 (1.6)	40 (21.7)	7 (12.1)
Invasive	12 (4.5)	0	22 (12.0)	5 (8.6)
Non-invasive	11 (4.1)	1 (1.6)	23 (12.5)	2 (3.4)
Parenteral nutrition	4 (1.5)	1 (1.6)	17 (9.2)	1 (1.7)
Duration of parenteral nutrition, days, mean (SD) [median]	4.5 (1.0) [5.0]	12.0 (-) [12.0]	8.6 (5.3) [7.0]	3.0 (-) [3.0]
Acute renal failure necessitating renal replacement therapy	1 (0.4)	0	5 (2.7)	1 (1.7)
Duration of renal failure, days, mean (SD) [median]	2.0 (-) [2.0]	-	14.3 (16.0) [9.5]	-
Septic shock during hospitalization	1 (0.4)	0	15 (8.2)	5 (8.6)
Mortality	2 (0.8)	0	1 (0.5)	3 (5.2)

*De-escalation of treatment to narrower-spectrum antibiotics upon patient improvement or confirmed microbiological diagnosis.

^†^Includes duration of all hospitalizations for patients with recurrences.

^‡^Does not include duration of all hospitalizations for patients with recurrences.

^§^Refers to patients hospitalized again (due to CAP) after initial discharge.

ICU = intensive care unit; SD = standard deviation.

## Discussion

The characteristics, clinical outcomes and hospital resource use of patients included in the REACH study were analysed according to their TCS using Halm’s criteria. Achieving early clinical stabilization (≤ 4 days) in patients with CAP appears to be associated with shorter hospital length of stay, lower incidence of admission to ICU or readmission to hospital for the same infection, and lower resource use compared with patients with a later response. The results presented here highlight how early identification of patients with a higher risk of a later response to treatment could assist the clinician in improving the chance of an early response by careful microbial stewardship, more aggressive treatment, early initiation of antibiotics and closer monitoring of response in these patients of concern.

Patients showing early or later response to treatment were similar in terms of demographics; however, there was a numerical tendency for those with a later response to have more comorbidity, or to have been in receipt of prior medication, in particular antivirals or antibiotics, in the 3 months prior to hospitalization. Data obtained from patients who were assessed for severity of disease with either the PORT/PSI or CURB-65 scoring systems suggest that patients with a later response had more severe disease than early responders. Intuitively it might make sense that patients with more severe disease will reach clinical stability later, and might require more aggressive treatment and closer follow-up, compared with patients who responded early to treatment. More patients with a later response had severity scores recorded than those with an early response. Moreover, use of severity scoring was variable or even absent in some countries (e.g. Belgium) and in those that did use a scoring system, PORT/PSI in particular, there was a trend to more patients being classed as moderate (III/IV) or high (V), which may suggest that physicians tend to score those patients with more severe disease.

The limitations associated with the retrospective design of this study, and the consequent unavailability of some values, have been discussed previously [3]. Significant heterogeneity in the use of Halm’s criteria for the assessment of clinical stability across the participating countries, ranging from 0% in Belgium, where TCS was not assessed using the Halm criteria in any patient, to 49.1% in the UK, and averaging only 28.7% overall, is in itself an important observation but highlights a limitation of this subanalysis. A higher mortality rate was observed in patients not assessed by Halm’s criteria. This may be because this sub-population had more severe disease according to the PORT/PSI and CURB-65 indices, but it is also possible that the use of Halm’s criteria indicates adherence to guidelines, and thus its use might be a surrogate of quality of healthcare.

Patients in both groups were treated largely following current ERS/ESCMID guidelines: aminopenicillin ± β-lactamase inhibitor ± macrolide; levofloxacin or moxifloxacin [4]. The observation that more early responders had been treated with amoxicillin–clavulanate monotherapy than later responders may be an indication that this treatment is recommended in local hospital guidelines for the treatment of CAP. In a recent study of treatment failure in patients hospitalized with CAP in Switzerland, it was found that those patients who had been treated initially with moxifloxacin or a β-lactam plus macrolide combination experienced lower treatment failure rates and reduced hospital stay, and thus reduced treatment costs, compared with patients receiving β-lactam monotherapy or a non-standardized antibiotic therapy [17].

Aliberti *et al*. [18] found that the interaction between host characteristics (immune status and comorbidities), pathogen characteristics (virulence, susceptibility and resistance to antibiotics) and antibiotic characteristics (timing, adequacy of therapy and pharmacokinetic factors) determines the time in which a patient reached clinical stability, and that the duration of IV therapy was, in most cases, found to be tailored to the patient’s clinical response. REACH was a retrospective observational study; thus, physicians were not required to report the decision-making process in the treatment of individual patients. However, the data obtained do illustrate the importance of identifying those patients of increased concern or at higher risk of being a later responder to treatment, such as patients with comorbidities. The high number of patients without a microbiological diagnosis indicates that treatment is, of necessity, empiric, with the potential for treatment failure, which impacts on clinical outcomes and healthcare resource use [19]. However, an understanding of the local epidemiology can inform the physician’s choice of treatment.

The higher frequency of initial antibiotic treatment modification due to insufficient response or treatment failure seen in the later responders group, compared with the early responders, may be explained by a higher incidence of inappropriate or discordant choice of initial antibiotic. It has been previously reported that the incidence of failure in patients with CAP ranges from 6 to 24% and may reach 31% in patients with severe CAP [13,20,21]. Factors most often associated with early failure have been reported to be more severe pneumonia, *Legionella* pneumonia, Gram-negative pneumonia, or mixed infections and discordant antimicrobial therapy [13,22]. Adherence or non-adherence to guidelines has been shown to be dependent on the hospital, and the speciality and training status of the prescribing physicians, and is thus an independent risk factor for treatment failure and mortality [22]. Empiric treatment that follows guidelines is associated with earlier TCS, and non-adherence may result in longer hospital length of stay and greater use of resources [23]. Data from the REACH study confirm that a longer TCS is associated with a longer length of hospital stay, and increased resource use such as more admissions to, and longer time in, ICU, mechanical ventilation, parenteral nutrition, acute renal failure and a higher incidence of home-based care after discharge, compared with patients with an early response.

In addition, recent data in patients with CAP have shown an association between a longer TCS and a significantly higher rate of adverse outcomes within 30 days of discharge compared with patients with a shorter TCS, which, the authors suggest, may be due to patients being discharged with a systemic inflammatory response [24]. In our study, later responders were found to have higher levels of reinfection/recurrence, compared with early responders. These observations reinforce the requirement for patients with a later response to treatment to receive closer clinical observation and management and a shorter time for follow-up visit after discharge. Our analysis of the effect of TCS on subsequent outcomes is slightly limited since some variables were not, or could not be, measured specifically before and after clinical stability. However, data on the length of hospitalization and on admissions to ICU after achievement of clinical stability show relatively small differences between early and later responders in this study and suggest such differences are more evident when looking at hospitalization as a whole and not at the post-clinical stability phase alone. In a recent retrospective analysis of the FOCUS comparative efficacy and safety trials of IV ceftaroline fosamil for the treatment of CAP [25], an advantage was seen versus the comparator ceftriaxone in response rates at Day 4 after initiation of treatment, using IDSA/ATS guidelines [5] for clinical stability.

In summary, this retrospective analysis of the data obtained in the observational REACH study of patients hospitalized with CAP, and with TCS assessment using Halm’s criteria, emphasizes the importance of an early response to treatment in terms of reduced morbidity and corresponding hospital resource use.

### Consent

Written informed consent was obtained from the patients for the publication of this report and any accompanying images.

## Competing interests

FB has received research grants from Chiesi, GlaxoSmithKline, Pfizer and Zambon, has received congress lecture fees from Abbott, Chiesi, GlaxoSmithKline and Pfizer and has received consultancy fees from AstraZeneca, GlaxoSmithKline and Pfizer.

HO has received research grants, speaking invitations and conference invitations from Astellas, AstraZeneca, Gilead, MSD, Pfizer and TEVA and consultancy fees from AstraZeneca, Gilead, MSD and TEVA.

JM and JR are employees of AstraZeneca.

KMB has received consultancy fees from ACT Oncology, AstraZeneca, BioSoteria, Celgene Corporation, Cypress Pharmaceuticals, Integrium LLC, Outcomes Research (now owned by Quintiles), MedImmune, Multiple Myeloma Research Foundation, Sigma-Tau Pharmaceuticals and Worldwide Clinical Trials.

JG has received research grants, speaking invitations and conference invitations from Astellas, AstraZeneca, Bayer, GlaxoSmithKline, Novartis, Pfizer and Vifor Pharma, and has recent or ongoing consultancies with Astellas, AstraZeneca, Bayer, Durata, GlaxoSmithKline, Janssen Cilag, Novartis, Pfizer, Theravance and Vifor Pharma.

## Authors’ contributions

The chief investigators (HO, FB, JG) designed the trial, with input from the sponsor. The chief investigators, together with KM, initiated the analysis presented here, with the other investigators, JM and JR contributing to the analysis and interpretation. The decision to submit the report for publication was made by the lead contributors and chief investigators, who drafted and finalized the report with the help of a medical writer. The sponsor funded editorial assistance and reviewed the draft before submission. All authors read and approved the final manuscript.
